# Comparing the Impact of Autologous Platelet-rich Plasma and Granulocyte Colony Stimulating Factor on Pregnancy Outcome in Patients with Repeated Implantation Failure

**Published:** 2019

**Authors:** Marzieh Mehrafza, Roya Kabodmehri, Zahra Nikpouri, Gholamreza Pourseify, Azadeh Raoufi, Azadeh Eftekhari, Sajedeh Samadnia, Ahmad Hosseini

**Affiliations:** 1- Mehr Fertility Research Center, Guilan University of Medical Science, Rasht, Iran; 2- Reproductive Health Research Center, Department of Obstetrics and Gynecology, Alzahra Hospital, Guilan University of Medical Sciences, Rasht, Iran

**Keywords:** Granulocyte colony-stimulating factor, Platelet-rich plasma, Repeated implantation failure

## Abstract

**Background:**

Despite the advancements in assisted reproductive technologies, repeated implantation failure (RIF) still remains a challenging problem for patients and clinicians. The aim of the present study was to compare the impact of intrauterine infusion of autologous platelet-rich plasma (PRP) and systemic administration of granulocyte colony stimulating factor (GCSF) on pregnancy outcome in patients with repeated implantation failure.

**Methods:**

The present retrospective cohort study included 123 patients with history of more than two repeated failed embryo transfers. Cycles were divided into two groups of intrauterine infusion of PRP (n=67) and systemic administration of GCSF (n=56). Pregnancy outcome was compared between two groups. The p-value less than 0.05 was considered statistically significant.

**Results:**

The clinical pregnancy rate was significantly higher in PRP group than GCSF group (40.3% versus 21.4%, p=0.025). The crud and adjusted odds ratios (95% confidence interval (CI)) were 2.5 and 2.6 (p=0.025, CI: 1.11–5.53 and p=0.03, CI: 1.10–6.15), respectively.

**Conclusion:**

It seems that intrauterine infusion of PRP can positively affect pregnancy outcome in RIF patients in comparison with systemic administration of GCSF and more studies need to be designed to conclude the effectiveness of this method.

## Introduction

Despite the developments in fertility treatment protocols, repeated implantation failure (RIF) still remains a challenging problem for patients and clinicians. RIF refers to failure in achieving clinical pregnancy following repeated embryos transfer. Various criteria for definition of RIF have been established but there is not any universal consensus on them (1–3).

Regarding factors influencing embryo implantation, many attempts have been developed for managing patients-including blastocyst transfer (4), assisted hatching (5), preimplantation genetic screening (6), hysteroscopy (7), removal of hydrosalpinges (8) and endometrial scratch (9). Also, there are some empirical treatments that some infertility specialist suggest for RIF patients. Intrauterine infusion of platelet-rich plasma (PRP) was first described by Chang et al. (10) in patients undergoing fertility treatment with thin endometrium. The effectiveness of PRP on induction of endometrial development has been also described by Zadehmodares et al. (11). A study by Nazari et al. (12) indicated that intrauterine infusion of PRP improves the pregnancy rate in RIF patients. A variety of cytokines and growth factors found in PRP includes transforming growth factor-β (TGF-β), platelet-derived growth factor (PDGF), insulin like growth factor-I (IGF-I), vascular endothelial growth factor (VEGF), epidermal growth factor (EGF), hepatocyte growth factor (HGF) and interleukin 8(IL-8) that promotes cellular migration, proliferation and differentiation processes (13).

Presence of granulocyte colony stimulating factor (GCSF) receptors in placental tissues, trophoblastic cells and endometrial cells indicate the importance of this cytokine in implantation (14–16). The use of GCSF in assisted reproductive technology (ART) has been reported by many studies to improve inadequate endometrium (17, 18). It also has been indicated that intrauterine (19) or systemic administration of GCSF can improve pregnancy rate in patients with RIF.

To the best of our knowledge, no studies have been found with the purpose of comparing the impact of PRP and GCSF administration on pregnancy rate of RIF patient in ART cycles. The present study evaluated the importance of PRP or GCSF administration in RIF patients to improve pregnancy outcome.

## Methods

The present retrospective cohort study was conducted at Mehr Fertility Research Center, a private medical institute, Rasht, Iran, during 2016–2017. One hundred twenty three patients with history of more than 2 repeated failed embryo transfer cycles were included in the study.

All instructions about the PRP and GCSF administration were given to all participants and following obtaining written consent, patients agreed to undergo intrauterine infusion of PRP or systemic administration of GCSF. All patients were recommended for PRP methods but they were free to choose one method according to their own financial or other conditions. Based on retrospective nature of our study, medical records of patients were studied and those undergone PRP (n=67) and GCSF (n=56) were recorded. All extracted data were compared between two groups.

All participants underwent basal hormonal screening, ultrasonography, hysterosalpingography and/or hysteroscopy. The pituitary was suppressed using gonadotropin releasing hormone (GnRH) agonist or antagonist. In patients undergoing GnRH agonist, depot decapeptyl 1.25 *mg* (Ferring, Germany) was administrated on the 21st day of previous cycle. In GnRH antagonist cycles, cetrotide 0.25 *mg* (Merck-Serono, Switzerland) were started daily when the leading follicle reached 12 *mm* in diameter. Ovarian stimulation was initiated with recombinant FSH (rFSH), and the daily dose of either rFSH (Gonal-F, Serono, Germany) or human menopausal gonadotropin (Menopur, Ferring, Germany) adjusted according to the ovarian response. Follicle development was monitored using transvaginal ultrasonography and estradiol measurements. Oocyte pick up was done 36 to 39 *hr* after triggering final oocyte maturation with human chorionic gonadotropin (hCG, Darou-Pakhsh, Iran). After denudation of oocyte-cumulus complexes, ICSI was performed. In fresh cycles, three to five days following ICSI procedures, up to three good and top quality embryos were transferred. Luteal phase was supported by 400 *mg* intravaginal (Aburaihan, Iran) and 100 *mg* intramuscular (Aburaihan, Iran) progesterone and 2.5 *mg* estrogen (Aburaihan, Iran).

In frozen embryo transfer (FET) cycles, endometrial preparation was started with 4 *mg/day* oral estradiol valerate (Aburaihan, Iran). Progesterone was started when a triple-line endometrial pattern and approximately thickness of 7 *mm* on ultrasound were seen. Embryos were transferred three to five days later, according to developmental stage of the embryos. All patients in PRP group and approximately half of GCSF group underwent FET cycles.

Two days before embryo transfer, peripheral venous blood (8.5 *ml*) was drawn into 10 *ml* syringe containing 1.5 *ml* anticoagulant solution. Manufacturer’s instruction was followed for preparing 1.5 *ml* lympho-PRP with platelet concentration 4–5 times higher than basal blood samples and 2000 *lymphocyte/μl* (Rooyagen, Iran). Intrauterine infusion of 1 *ml* lympho-PRP was performed with intrauterine insemination catheter.

In GCSF group, patients were treated with a single administration of 300 *μg* recombinant GCSF (PD-Grastim, Pooyesh Darou, Iran), two hours before embryo transfer.

Chemical pregnancy was confirmed by positive βhCG test, 14 days after embryo transfer. Clinical pregnancy was determined after ultrasound observation of fetal heart at 7^th^ weeks of pregnancy.

### Statistical anabasis:

Normal distributed variables were analyzed using student’s t-test. If data was not normalized with log transformation, Mann-Whitney test was used. Chi-square test was used for categorical variables. Crude and adjusted odds ratios were reported using logistic regression. According to univariate logistic regression, variables with p-value less than 0.2 were considered confounding and evaluated by multivariate logistic regression (Backward method). Statistical analysis was done using statistical package for the social sciences version 23 (SPSS Inc. Chicago, IL, USA). P-value less than 0.05 was considered statistically significant.

## Results

The mean age of patients was 32.57±5.23. The baseline characteristics of patients are presented in table 1. Numbers of previous embryo transfer cycles were significantly higher in PRP group (p=0.01).

**Table 1.** The baseline characteristics of patients

**Variables**	**PRP group (n=67)**	**GCSF group (n=56)**	**p-value**
**Age (years)**	31.85±5.22	33.46±5.17	0.11^*^
**BMI (*kg/m^2^*)**	25.52±3.47	26.44±3.61	0.24^*^
**LH (*mIU/ml*)**	3 (0.1–15)	2.95(0.3–8)	0.96^**^
**FSH (*mIU/ml*)**	4.59±1.71	5.29±2.18	0.06^*^
**AMH (*ng/ml*)**	3.02±1.85	2.08±2.59	0.06^*^
**Number of previous embryo transfer cycles**	3(2–9)	2(2–5)	0.01^**^

* T-test,

** Mann-Whitney test,

*** Chi-square test.

Data are presented as mean±standard deviation, median (Minimum-maximum) and percentage. BMI: Body Mass Index, LH: Luteinizing Hormone, FSH: Follicular Stimulating Hormone. AMH: Anti-Mullerian Hormone

The stimulation characteristics and pregnancy outcome of patients are presented in table 2. Patients with GnRH agonist protocol, mean number of oocyte retrieved and metaphase II were significantly higher in PRP group than GCSF group (p=0.03, p=0.02 and p=0.024, respectively).

**Table 2.** The stimulation characteristics and pregnancy outcome

**Variables**	**PRP group**	**GCSF group**	**p-value**
**Type of GnRH analogue**			
Agonist (%)	56 (87.5)	40 (71.4)	0.03 ^**^
Antagonist (%)	8 (12.5)	16 (28.6)	
**Total gonadotropin dose (*IU*)**	2365.83±1.5	2273±1.6	0.62 ^*^
**Total number of oocyte retrieved**	13.62±7.02	10.8±5.64	0.02 ^*^
**Metaphase II**	10.75±6.48	8.32±4.85	0.024 ^*^
**Total number of embryos**	6.65±1.94	5.24±2.16	0.07 ^*^
**Fertilization rate (%)**	500/858(58.3)	376/605(62.1)	0.4 ^*^
**Number of embryos transferred**	2.74±0.86	2.61±0.95	0.45 ^*^
**Number of blastocyst transferred**	0.73±0.93	0.79±1	0.76 ^*^
**Implantation rate (%)**	33/204 (17.2)	15/143 (10.5)	0.14 ^*^
**Chemical pregnancy (%)**	29/67 (43.3)	15/56 (26.8)	0.057 ^**^
**Clinical pregnancy (%)**	27/67(40.3)	12/56 (21.4)	0.025 ^**^

* T-test,

** Chi-square test.

Data is presented as mean±standard deviation and percentage

The chemical and clinical pregnancy rate was 43.3% and 40.3% in PRP group and 26.8% and 21.4% in GCSF group (p=0.057, p=0.025, respectively). The univariate logistic regression indicated that age (p=0.11), frozen-thawed embryo transfer (p=0.02) and type of GnRH analogue (Agonist or antagonist) (p=0.09) had p-value less than 0.2. Results of univariate and multivariate logistic regression are summarized in table 3. Backward method of multivariate logistic regression indicated that patients who underwent PRP had significantly improved outcome as compared to patients underwent GCSF.

**Table 3.** The crud and adjusted odds ratios of clinical pregnancy for confounding variables (Backward method)

**Confounding variables**	**Univariate logistic regression**			**Multiple logistic regression**		

	**p-value**	**Odds ratio**	**95% CI**	**p-value**	**Odds ratio**	**95% CI**
**Step 1 ^a^**						
Frozen-thawed embryo transfer	0.02	3.6	(1.27,10.15)	0.39	1.75	(0.50,6.17)
Age	0.11	0.94	(0.87,1.02)	0.37	0.96	(0.88,1.05)
Type of GnRH analogue	0.09	0.37	(0.12,1.15)	0.26	0.5	(0.15,1.67)
PRP method	0.025	2.50	(1.11,5.53)	0.27	1.76	(0.64,4.83)
Constant				0.9	0.82	
**Step 4 ^a^**						
PRP method				0.03	2.6	(1.10,6.15)
Constant				0.000	0.25	

a: Variable(s) entered on step 1: Frozen-thawed embryo transfer, Age, Type of GnRH analogue, PRP method

## Discussion

PRP is a part of autologous plasma that has platelets higher than baseline concentration. Platelets store various growth factors and cytokines in their cytoplasmic granules that undergo exocytosis in the presence of activating factors such as collagen of extracellular matrix (20). PRP is prepared from peripheral blood without risk of viral infection and immunological reactions.

Based on leukocyte and fibrin content, there are four categories of PRP; pure platelet-rich plasma (P-PRP), leukocyte- and platelet-rich plasma (L-PRP), pure platelet-rich fibrin (P-PRF) and leukocyte and platelet-rich fibrin (L-PRF) (21). PRP has been used in gynecological disorders including Asherman syndrome management (22) symptomatic vaginal mesh exposure (23), wound healing after cesarean section (24), treatment of thin endometrium following hormone therapy for embryo transfer (10) and premature ovarian failure (25).

Srivastava et al. (26) indicated that during implantation process and in response to hCG produced by pre-implantation embryos, endometrial epithelial and stromal cells synthesize many cytokines and growth factors including IFNG, IL-1b, IL-6, TNF, IL-8, PDGF, TNF and VEGF acts locally and influences different mechanisms like inflammation, invasion, differentiation, proliferation and cell adhesion. Through activating platelets of PRP, various cytokines and growth factors, necessary for enhancing endometrial receptivity and improving implantation rate, are secreted (27). Implantation process is a consequence of inflammatory and anti-inflammatory equilibrium which imbalance state of these mechanisms may result conditions like RIF (28). So, it seems that one possible mechanism of PRP action on receptivity of endometrium is via anti-inflammatory action of factors like HGF (29).

There are some reports that affirm the endometrial imbalance expression of immune factors in RIF patients (30–32). In order to overcome this abnormal profile, many strategies have been developed. The effect of intrauterine administration of cultured peripheral blood mononuclear cells prior to embryo transfer on pregnancy outcome has been described in RIF patients (33, 34). Moreover, there are some reports that affirm the efficiency of PRP for treatment of RIF patients (12, 35, 36).

The benefit of intrauterine or systemic administration of GCSF regarding pregnancy outcome in RIF patients has been reported in some studies (19, 37). GCSF is produced by various types of cells including fibroblasts, endometrial and natural killer cells (38, 39). The impacts of GCSF on ex-vivo expression of key endometrial genes involving implantation process have been proved (14).

Wurfel et al. (37) indicated that systematical administration of GCSF has positive effects on patients who have killer cell immunoglobulin-like receptor (KIR) genes deficiency. Indeed, the interaction between human leukocyte antigen (HLA-C) ligand from embryonic trophoblast and KIRs from uterine natural killer (uNK) cells has been disordered in these patients.

Salmassi et al. (40) reported the predictive potency of serum GCSF level for IVF outcome. The influence of GCSF on pregnancy rate of RIF patients has been evaluated. In a clinical trial by Davari-Tanha et al. (41), significant improvement in the rate of implantation and chemical pregnancy was reported in RIF patients undergone 300 *μg/ml* G-CSF compared with saline and placebo group but clinical pregnancy rate was not affected. In a study by Aleyasin et al. (42), subcutaneous administration of GCSF in patient with RIF improved the rate of implantation, chemical and clinical pregnancy in comparison with patients who did not receive. The clinical pregnancy rate was significantly higher in GCSF group than control (37.5% vs. 14.3%, p=0.005).

In our study, the impact of intrauterine L-PRP on ICSI outcome was assessed in non-randomized RIF patients as compared to subjects who received systemic GCSF. The retrospective nature of the present study made it dependent on control confounding factors. It was indicated that patients undergoing PRP had significantly improved outcome as compared to patients undergoing GCSF, although pregnancy outcome may be affected by other factors. In particular, the clinical pregnancy rate was adjusted for variables with p-value less than 0.2 (Age, frozen-thawed embryo transfer and type of GnRH analogue) as univariate logistic regression has indicated. When confounding variables were included to logistic regression model, PRP method significantly increased pregnancy rate.

There are two limitations with respect to the present study. The first is the observational nature of the study that is not a randomized clinical trial. The results of the present study indicated a significantly higher clinical pregnancy rate in patients undergoing PRP and after adjusting of confounding factors, the superiority of PRP method compared to GCSF was also approved.

The second is the lack of control group. According to internal policy of our institute, with respect to detected maternal or embryonic factors, all patients with more than two implantation failures took part in some clinical approaches, so no RIF patients could be found, nullifying the need for control group.

## Conclusion

The results of the present study indicated that PRP method may be beneficial for RIF patients. Although we attempted to control the impact of confounding factors, more blinded randomized clinical trials should be designed to confirm the efficiency of PRP method. The mechanisms by which PRP affects pregnancy rate remain unclear and more investigations are needed.
